# Keeping Elo alive: Evaluating and improving measurement properties of learning systems based on Elo ratings

**DOI:** 10.1111/bmsp.12395

**Published:** 2025-06-06

**Authors:** Maria Bolsinova, Bence Gergely, Matthieu J. S. Brinkhuis

**Affiliations:** ^1^ Department of Methodology and Statistics Tilburg University Tilburg The Netherlands; ^2^ Institute of Psychology Károli Gáspár University of the Reformed Church in Hungary Budapest Hungary; ^3^ Department of Information and Computing Sciences Utrecht University Utrecht The Netherlands

**Keywords:** adaptive learning systems, Elo rating system, measurement

## Abstract

The Elo Rating System which originates from competitive chess has been widely utilised in large‐scale online educational applications where it is used for on‐the‐fly estimation of ability, item calibration, and adaptivity. In this paper, we aim to critically analyse the shortcomings of the Elo rating system in an educational context, shedding light on its measurement properties and when these may fall short in accurately capturing student abilities and item difficulties. In a simulation study, we look at the asymptotic properties of the Elo rating system. Our results show that the Elo ratings are generally not unbiased and their variances are context‐dependent. Furthermore, in scenarios where items are selected adaptively based on the current ratings and the item difficulties are updated alongside the student abilities, the variance of the ratings across items and students artificially increases over time and as a result the ratings do not converge. We propose a solution to this problem which entails using two parallel chains of ratings which remove the dependence of item selection on the current errors in the ratings.

## 1 INTRODUCTION

The Elo Rating System (ERS), devised by Árpád Élő in the mid‐20th century (Batchelder & Bershad, 1979; Elo, 1978), has established itself as a popular algorithm for evaluating player performance in various competitive activities, starting with chess and extending to other sports, animal science, plant breeding, computer security, and many more (e.g., Albers & de Vries, 2001; Hvattum & Arntzen, 2010; Pieters et al., 2012; Simko & Pechenick, 2010). Educational practice and online learning environments apply such systems to dynamically measure student ability and item difficulty. Pelánek (2016) already lists several educational applications of the ERS from before 2016. Since then, many more applications have appeared, including measuring task difficulty (Pankiewicz, 2020), recommending coding exercises (Mangaroska et al., 2019), game‐based assessment (Ruipérez‐Valiente et al., 2022), design of assessment task analytics dashboards (Keskin et al., 2024), skill taxonomy applications (Yudelson et al., 2019), adaptive practice of facts (Pelánek et al., 2017), and modelling student knowledge in blended learning (Hamzah & Sosnovsky, 2023).

The popularity of the ERS in large‐scale educational applications is partly explained by its simplicity and computational efficiency. After every person‐item interaction, the person and item ratings are updated using a simple formula (Klinkenberg et al., 2011; Pelánek, 2016):

(1) $${\hat{\theta }}_{j(t+1)}={\hat{\theta }}_{jt}+K(X_{jit}-ℰ(X_{jit}));$$

(2) $${\hat{\delta }}_{i(t+1)}={\hat{\delta }}_{it}-K(X_{jit}-ℰ(X_{jit})),$$

where ${\hat{\theta }}_{jt}$ and ${\hat{\delta }}_{it}$ are ability and difficulty ratings of person j and item i, respectively, K is the value determining the size of the update, $X_{jit}$ is the observed response of person j to item i at timepoint t (1 – correct, 0 – incorrect), and $ℰ(X_{jit})$ is it expected value calculated using the current ratings:

(3) $$\mathrm{Pr}(X_{jit}=1)=\frac{\exp({\hat{\theta }}_{jt}-{\hat{\delta }}_{it})}{1+\exp({\hat{\theta }}_{jt}-{\hat{\delta }}_{it})}.$$

As such, the standard Elo algorithm can be seen as a way of estimating dynamically changing parameters of the Rasch (1960) model.^1^

While many of the applications of ERS are low‐stakes and require just approximate estimates of player performance, educational applications require more precise measurement properties. For example, student performance is used for communicating progress and matching practice material with a given difficulty (e.g., Jansen et al., 2016), reporting to teachers and parents, suggesting other domains to play (Brinkhuis et al., 2020), etc. In more serious educational applications, the measurement properties of ratings become increasingly important. Two major properties to focus on are bias and variance.

Properties of Elo ratings have been studied analytically in simplified scenarios before. For example, Avdeev (2015) investigated the convergence properties of Elo when a player competes against a single adversary, Zanco et al. (2024) provided a comprehensive study of the properties of Elo ratings in round‐robin tournaments and Jabin and Junca (2015) showed that Elo ratings converge to their true values when there is an infinite number of players competing against each other, but when players compete only with other players of similar strength, convergence is problematic. However, it is not yet clear what the precise properties of the ERS are in learning scenarios. Specifically, in finite systems when we have two pools of players (one for the persons and one for the items) instead of a single pool, and when item selection is adaptive, based on the current values of the ratings. Previous research has shown that Elo has some inherent limitations in this situation. For example, Brinkhuis and Maris (2009, 2010) demonstrate the lack of stationarity of the distribution of Elo ratings in certain situations, and Hofman et al. (2020) show that the variance of ratings inflates in certain adaptive scenarios. We posit that the measurement properties of the ERS have not been sufficiently evaluated for learning scenarios, a domain where stakes can be high and the bias and variance of estimators should be studied. In addition, to our knowledge, no fundamental improvements to the ERS have been suggested in this domain. Therefore, we aim to critically analyze the shortcomings of the ERS in an educational measurement context, shedding light on its properties and when these may fall short in precisely and reliably capturing student abilities and item difficulties. In addition, to keep Elo alive in the context of educational measurement and learning applications, we propose directions for improving the ERS.

The rest of the paper is organised as follows. In Section 2, we present a simulation study to evaluate the measurement properties of Elo ratings when the item and person parameters are stable. Section 3 focuses on one of the main findings of this simulation, namely that when items are selected adaptively and calibrated on the fly, then the variance of the Elo ratings (across persons and items) artificially increases over time. We explain why this problem occurs and propose a solution to it. In Section 4, the proposed modification is evaluated in an additional simulation study. It is also compared to another rating system, the Urnings (Bolsinova, Brinkhuis, et al., 2022; Bolsinova, Maris, et al., 2022), which was specifically designed to have desirable measurement properties. The paper ends with a discussion.

## 2 PROPERTIES OF THE ELO RATINGS

To demonstrate the properties of Elo ratings we performed a simulation study^2^ in which we looked at what happens to the ratings when students solve a large number of items in a system and all true item and person parameters are stable.

### 2.1 Simulation setup

We investigated the asymptotic properties of the ratings to answer the following questions: (1) Do the Elo ratings always have invariant distributions (i.e., do the ratings of each person reach a stable level around which they fluctuate)? (2) If an invariant distribution exists, is its mean different from the true value (i.e., are the ratings unbiased), and (3) what is its variance? (4) How much data is needed for the ratings to reach their invariant distribution if it exists? (5) How do the properties of the invariant distribution depend on the person's ability level in relation to the item pool? (6) How do these properties of the Elo ratings depend on how the system is set up? Here, we consider two factors. First, item parameters are either fixed at pre‐calibrated values or updated on the fly together with the person parameters (i.e., fixed vs. updated items). Second, item selection is either random or conditional on the current values of the person's rating and the item ratings (i.e., random vs. adaptive item selection). The combination of these two factors results in a 2‐by‐2 design with four simulation scenarios (fixed‐random, fixed‐adaptive, updated‐random, and updated‐adaptive).

In each condition, we used a single set of true values for θs and δs and repeatedly simulated data from a learning system. In each of the 500 replications of the system, at each of the 1,000 timepoints, we generated a response to an item (either randomly or adaptively selected from a pool of 200 items) for each of the 1,000 persons. The responses were generated under the Rasch model (see Equation 3) using the true values (constant across timepoints). After every response, the Elo ratings were updated according to Equations 1 and 2. In the scenarios with fixed items, only abilities were updated, while the difficulties were fixed to their true values.^3^

With such a setup, for each person, we have 500 replications of their Elo ratings at each of the 1000 timepoints, and hence we can look at the distribution of the Elo ratings (across replications) for different timepoints. We calculated the means and the variances of these distributions. If these means and variances stabilize with time, then it means that the ratings reach their invariant distributions, and the corresponding means and variances are the estimates of the means and variances of the invariant distributions of these persons. To decrease the effect of sampling variability, we averaged these person‐specific means and variances across the last 500 timepoints. We evaluated the (absolute) bias of the Elo ratings by computing the (absolute) difference between the mean of the invariant distribution and the true value. If the ratings reached an invariant distribution, to evaluate how fast the ratings converge, we calculated a hitting time (i.e., the timepoint at which the mean of each person's rating reaches, for the first time, within a 0.01 range of the invariant mean).

In all scenarios, the true δs were equal to equally spaced quantiles of $N(\delta_{i};0,1.5)$. The true θs were equally spaced quantiles of $N(\theta ;\mu_{\theta },1)$. The standard deviation of the item distribution was 1.5 times larger than that of the person distribution to ensure that for each person there is a sufficient number of items above and below them on the scale. In non‐adaptive scenarios $\mu_{\theta }=\ln(.7/(1-.7))$ to mimic a system with relatively easy items (i.e., the probability of a correct response of an average person to an average item is .7). In adaptive scenarios $\mu_{\theta }$ depended on the item selection setting (see details below). The starting values for the $\hat{\delta }$s were set to 0. The starting values of $\hat{\theta }$s were set to $\mu_{\theta }$ to ensure that all conditions had the same distances between the starting and true values making the hitting times directly comparable.

In the adaptive scenarios, item selection was done by sampling from a multinomial distribution proportional to a normal density:

(4) $$\mathrm{Pr}(I_{jt}=i)=\frac{N\left({\hat{\theta }}_{jt}-{\hat{\delta }}_{it};\ln\left(\frac{p}{1-p}\right),0.5\right)}{\sum_{k=1}^{200}N\left({\hat{\theta }}_{jt}-{\hat{\delta }}_{kt};\ln\left(\frac{p}{1-p}\right),0.5\right)}$$

where $I_{jt}\in \{1,\text{…},200\}$ is the indicator for the item selected for person j at iteration t, and p is the desired probability of a correct response. Items with the expected probability of a correct response equal to p have the highest selection probability, while the standard deviation of 0.5 around the mean allows for some variability in the difficulty of the selected items.

In each scenario, we defined a baseline condition to which other conditions were compared: K=0.3 and p=.7 (for adaptive scenarios). Next, we included conditions with K of 0.1, 0.2, 0.4, and 0.5 (while keeping p=.7). Finally, in the adaptive scenarios, the effect of the adaptivity rule was investigated by including conditions with p of .5, .6, .8, and .9 while keeping K=0.3. Here, we set $\mu_{\theta }=\ln(p/(1-p))$ to ensure that the conditions are comparable in terms of how well the adaptivity rule matches the relationship between the persons and the items.

Since the ability and difficulty parameters are only interpretable in relation to each other, an identification constraint for the ratings is required in the updated‐random and updated‐adaptive scenarios. The default constraint of the Elo ratings (i.e., their sum is constant and equal to the sum of the starting values) does not provide an intuitive interpretation. Instead, we chose to use the mean of the item difficulties as a reference point, that is, we focus on the differences between ability/difficulty and the average item difficulty. Operationally, after finishing the simulation at every timepoint we subtracted the mean of the item ratings from all ratings (i.e., identification constraint $\sum_{i}{\hat{\delta }}_{it}\equiv 0$ was enforced), which ensures that after this transformation at every timepoint ${\hat{\theta }}_{jt}$ can be interpreted as the difference from the item mean.

### 2.2 Simulation results

**FIGURE 1:**
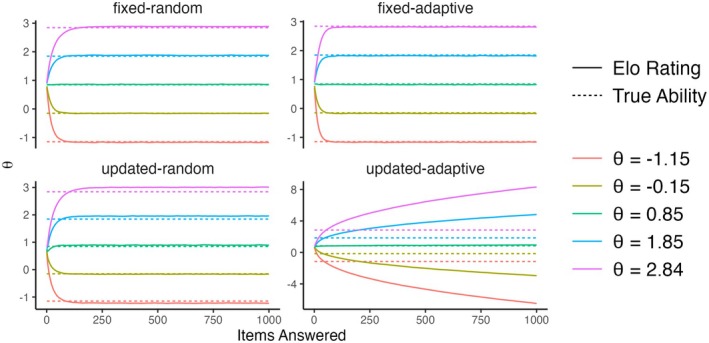
The means of the Elo ratings of five persons with different true values of ability (θ) at different timepoints in the baseline conditions of the four simulation scenarios.

**FIGURE 2:**
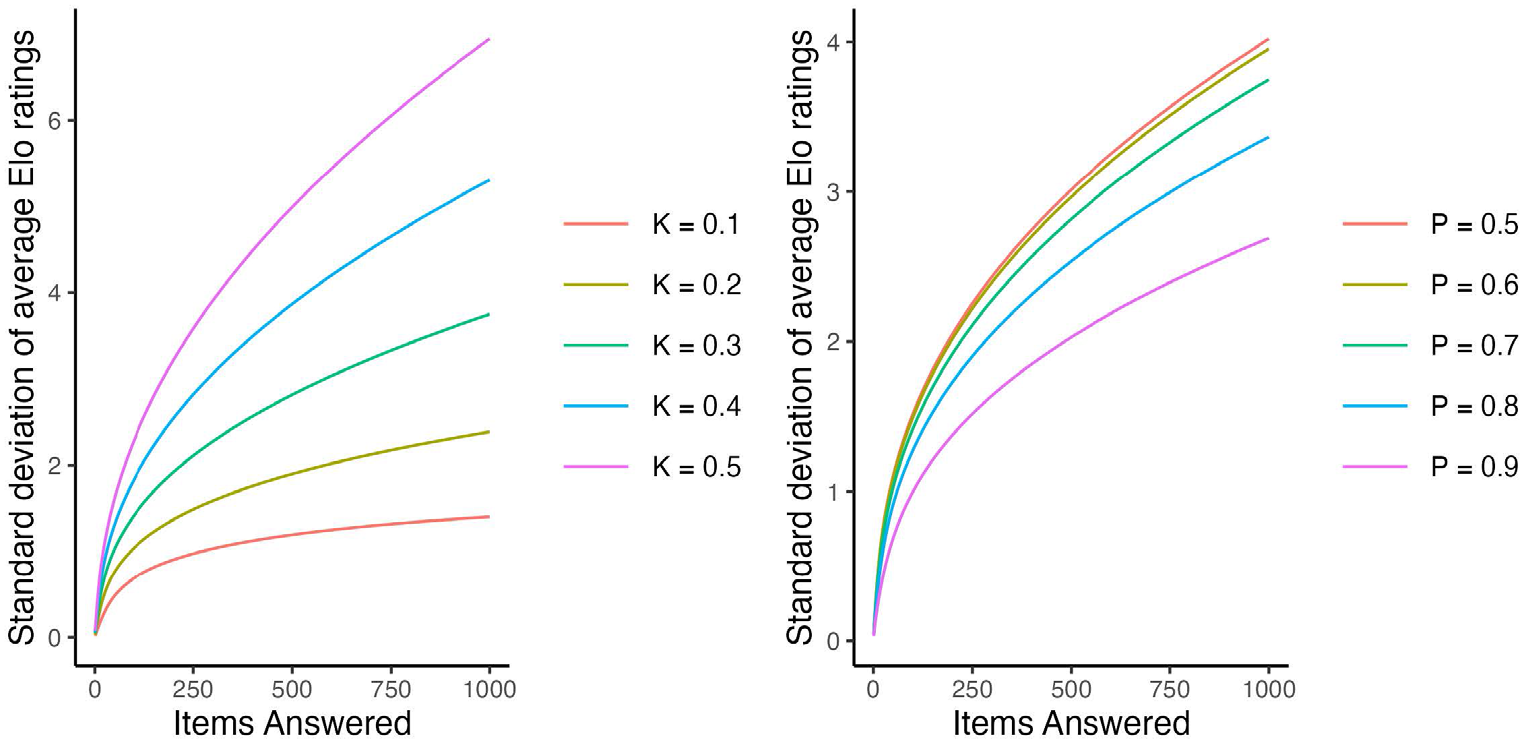
Severity of variance inflation of the Elo ratings in the updated‐adaptive scenario depending on the K‐value (on the left) and on the desired probability of a correct response (p, on the right).

First, we consider the results of the baseline conditions.^4^ Figure 1 shows for five persons (with θ closest to $\mu_{\theta },\mu_{\theta }\pm 1$, and $\mu_{\theta }\pm 2$) how the mean of their ratings (across replications) changes across time in the four scenarios. In the first three scenarios, after moving away from the starting values, the means stabilize. That is, the ratings have an invariant distribution. In all three scenarios, while the means of the invariant distributions are very close to the true values (indicated by dashed lines), they are not exactly equal to them. In the updated‐adaptive scenario, however, the ratings do not converge to an invariant distribution, but rather diverge away from each other (see bottom‐left panel). This is a phenomenon called rating variance inflation (Bolsinova, Brinkhuis, et al., 2022; Hofman et al., 2020), meaning that the variance of the ratings across persons increases over time. The severity of this inflation depends on K: stronger for larger values, see Figure 2 on the left, and p: strongest for p=.5, see Figure 2 on the right.

**FIGURE 3:**
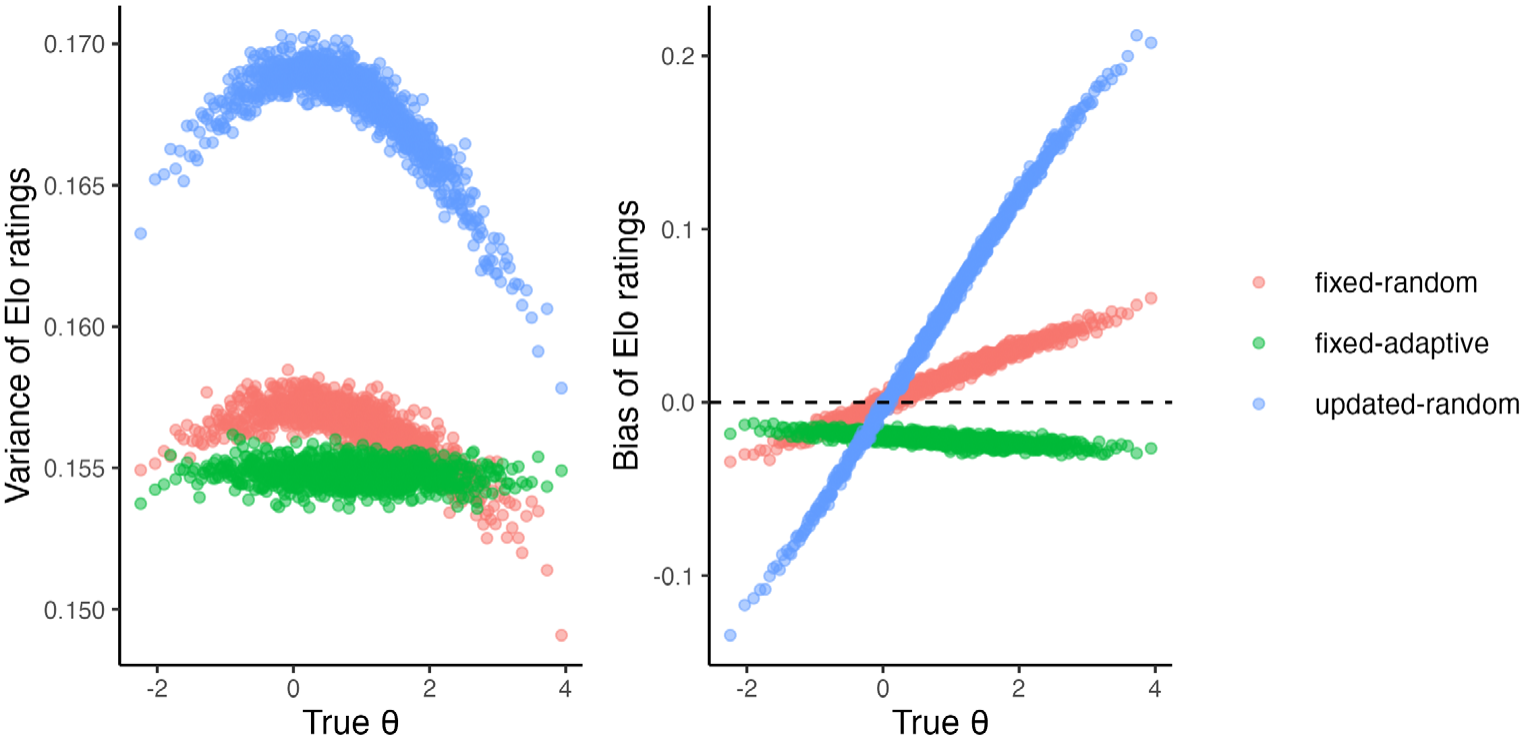
Bias and Variance of the Elo ratings as the function of the true values of ability (θ) in the baseline conditions in the three simulation scenarios where the Elo ratings have an invariant distribution.

Now we discuss the properties of the invariant distribution for those scenarios where the ratings converge. In Figure 3, the difference between the invariant means of the ratings and the true values (i.e., the bias of the Elo ratings) is plotted against the true θs. In the fixed‐random and updated‐random scenarios, for the persons with θ>0 (i.e., above the average item difficulty), ability is overestimated, while for the persons with θ<0 it is underestimated, that is, outward bias is present. Different from the random scenarios, in the fixed‐adaptive scenario all ratings are negatively biased in the baseline condition (see Figure 3 on the right in green).

**FIGURE 4:**
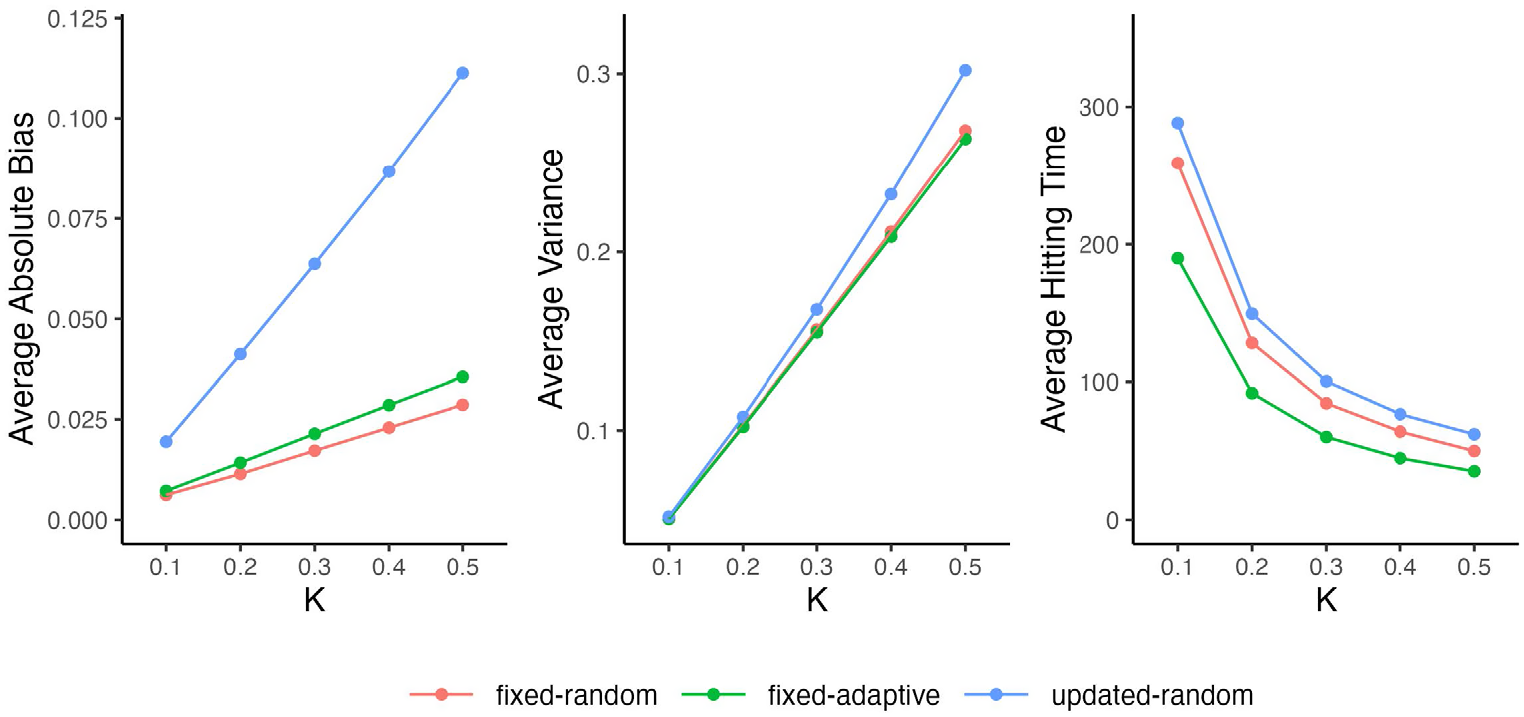
Effect of the K value on the average absolute bias (left), average variance (middle) and average hitting times (right) of the Elo ratings in the three simulation scenarios where the Elo ratings have an invariant distribution.

**FIGURE 5:**
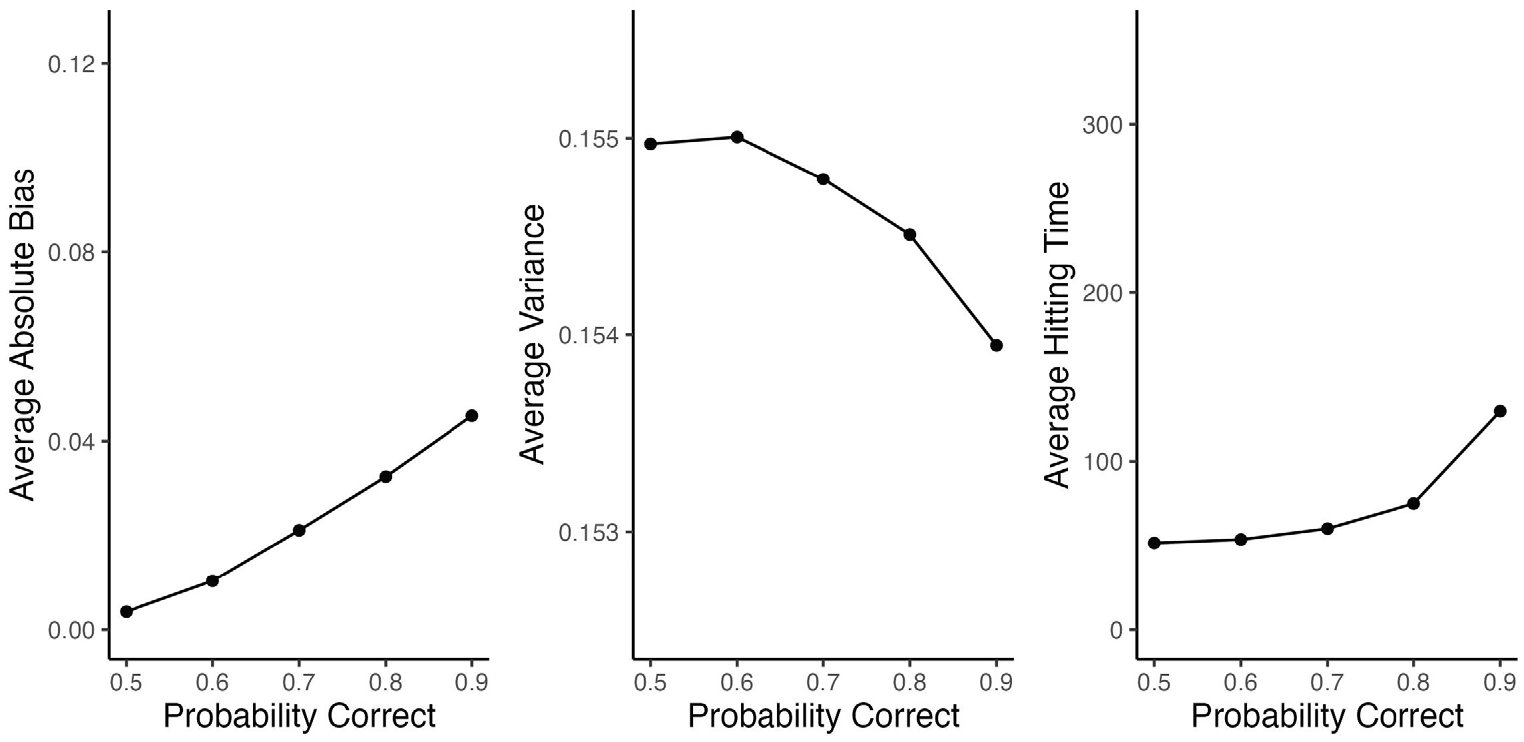
Effect of the desired probability of a correct response (p) on the average absolute bias (left), average variance (middle) and average hitting times (right) of the Elo ratings in the fixed‐adaptive scenario.

When different K‐values are used, the ratings also converge in the first three scenarios and the magnitude of bias increases with K (see Figure 4 on the left). Furthermore, in the fixed‐adaptive scenario bias depends on the desired probability of a correct response (see Figure 5 on the left). For p=.5 there is hardly any bias, and the magnitude of bias increases when P moves away from .5. The absolute bias is on average slightly higher in the fixed‐adaptive scenario than in the fixed‐random scenario, and significantly higher in the updated‐random scenario.

Figure 3 (on the left) shows how the variances of the invariant distributions of the Elo ratings depend on the true ability. In the random scenarios, the variance is the highest when the ability is close 0 (i.e., to the average item difficulty), and it decreases when moving away from it. This is different from the standard errors of expected a posteriori estimates of ability which are often used in item response theory in testing applications, where the errors are the lowest when the ability is around the average item difficulty and uncertainty increases for more extreme levels of ability. However, it is not unexpected because the average size of the steps (${\hat{\theta }}_{j(t-1)}-{\hat{\theta }}_{jt}$) is the highest when ℰ(X)=.5. In the fixed‐adaptive scenario, the ratings' variances hardly depend on the true values. This is because the proportion of correct responses is roughly the same across persons with only small deviations in the tails where it might deviate from p due to there not being many items of desired difficulty. The variances are generally higher in the updated‐random scenario compared to the fixed scenarios. The variance of the ratings increases with K (see Figure 4 in the middle), but hardly depends on p in the fixed‐adaptive scenario (see Figure 5; for p=.9 it is around .001 smaller than for p=.5).

While in all conditions of the first three scenarios, the ratings reach an invariant distribution, how fast it happens depends on the K‐value. Doubling K results in roughly halving the hitting time (see the differences between K=0.1 and K=0.2, and between K=0.2 and K=0.4 in Figure 4 on the right). In the fixed‐adaptive scenario convergence also depends on p (slower for more extreme p, see Figure 5on the right). Increasing p from .7 to .9 results in roughly 50% more responses needed to converge, which is similar to decreasing K from .3 to .2. That is, while p does not have a direct effect on the variance of the ratings, to retain the speed of convergence when presenting students with easier items, the K‐value should increase leading to an increase of variance (i.e., a decrease in measurement precision and reliability). Convergence is on average the fastest in the fixed‐adaptive scenario, followed by the fixed‐random and the updated‐adaptive scenarios.

## 3 FIXING VARIANCE INFLATION

Abnormal behaviour in the updated‐adaptive scenario is highly problematic because the use of Elo is especially advocated when on‐the‐fly item calibration is needed and when the main purpose of ability measurement is to optimize item selection. Therefore, it is important to study why rating variance inflation occurs and try to remove it.

### 3.1 Why does rating variance inflation occur?

Let us have a closer look at why the Elo ratings drift away from each other in the updated‐adaptive scenario. Hofman et al. (2020) and Bolsinova, Maris et al. (2022) provided a general explanation that when in a Markov chain the transition kernel is selected conditional on the current values, the chain would not converge to the same invariant distribution which it would have if any of these kernels individually or their random combination were used. But how exactly does it apply to the chain of Elo ratings?

**FIGURE 6:**
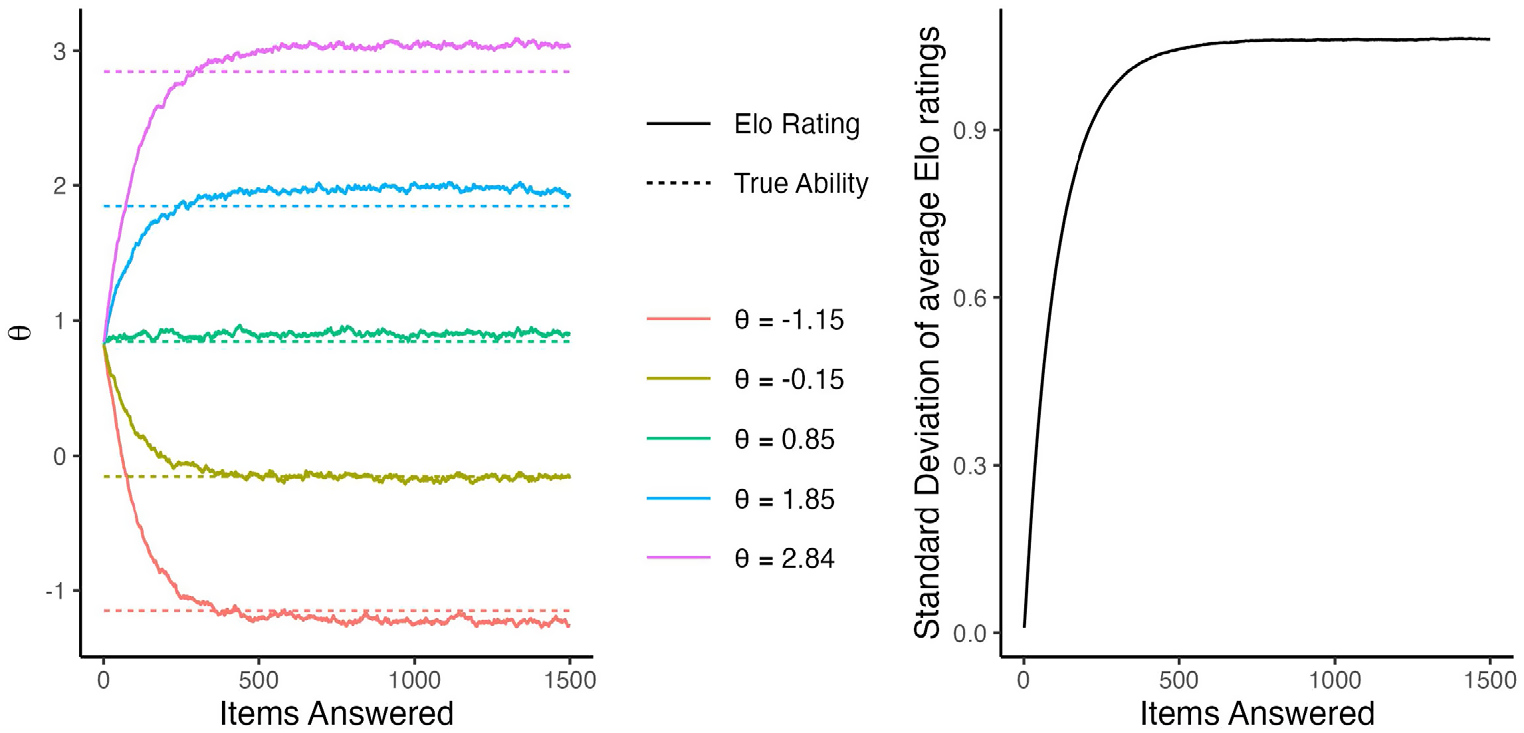
Average Elo ratings (on the left) and standard deviation of the average Elo ratings (on the right) over time when item selection is based on the true values of ability and difficulty.

Importantly, rating variance inflation would not occur if instead of using the ratings, one could use the true values for item selection (see Figure 6). Hence, variance inflation happens not because students with higher ability receive more difficult items, while students with lower ability receive easier items, but rather because item selection depends on the current value of the errors (differences between the ratings and the true values). Let us inspect these errors. When items are updated alongside the persons, at every timepoint some of them are (slightly) overestimated (i.e., ${\hat{\delta }}_{it}>\delta_{i}$), while other items are (slightly) underestimated (i.e., ${\hat{\delta }}_{it}<\delta_{i}$). For each person j at each timepoint t let us consider the expected value of the error of the selected item:

(5) $${ℰ}_{jt}({\hat{\delta }}_{t}-\delta )=\underset{i}{\sum }({\hat{\delta }}_{it}-\delta_{i})\mathrm{Pr}(I_{jt}=i\;|\;{\hat{\theta }}_{jt},{\hat{\delta }}_{t}).$$

**FIGURE 7:**
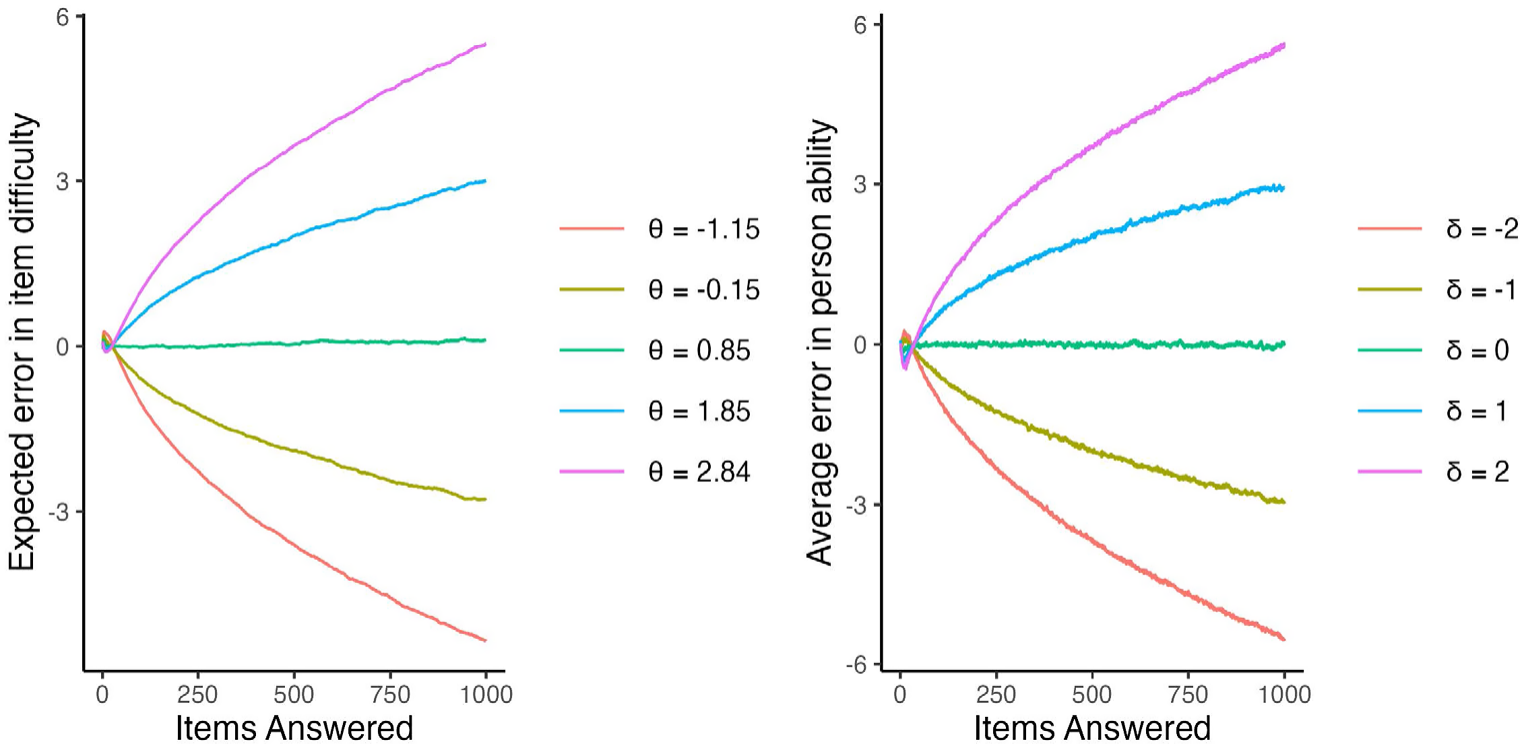
Expected error in the item difficulties of selected items for persons with different levels of ability (on the left) and average error in the abilities of the persons for whom items with different difficulty are selected (on the right) over time in the baseline condition of the updated‐adaptive scenario.

When the items are selected uniformly, then the expected error is equal to zero since $\sum_{i}\delta_{i}\equiv \sum_{i}{\hat{\delta }}_{it}\equiv 0$. But when item selection is adaptive, then for persons with high θ the majority of the items are more likely to be selected when they are overestimated than when they are underestimated, therefore the expected error in Equation 5 is positive. Analogously, for persons with low θ this expected error is negative. Figure 7 (on the left) illustrates this for the baseline condition of the updated‐adaptive scenario. Over time, this pattern reinforces itself and becomes stronger.

A similar pattern is present on the item side. For difficult items the average difference between the rating of the person for which this item is selected and their true value is positive, that is, difficult items are more often presented to persons who are at that timepoint overestimated. Analogously, easy items are more often presented to persons who are at that timepoint underestimated. To illustrate this, for each item i in each replication, we computed $\left({\hat{\theta }}_{jt}-\theta_{j}\right)$ for the last person at timepoint t for whom it was selected. Figure 7 (on the right) shows that these values (averaged across replications) increase over time for difficult items and decrease for easy items. Hence, the same as on the person side, this pattern reinforces itself and becomes stronger over time. In the fixed‐adaptive scenario, difficult items are also more often presented to overestimated persons, rather than to underestimated persons (and vice versa for easy items), but this does not lead to rating variance inflation because the scale is fixed through the items.

### 3.2 Possible solution: Parallel Elo

**FIGURE 8:**
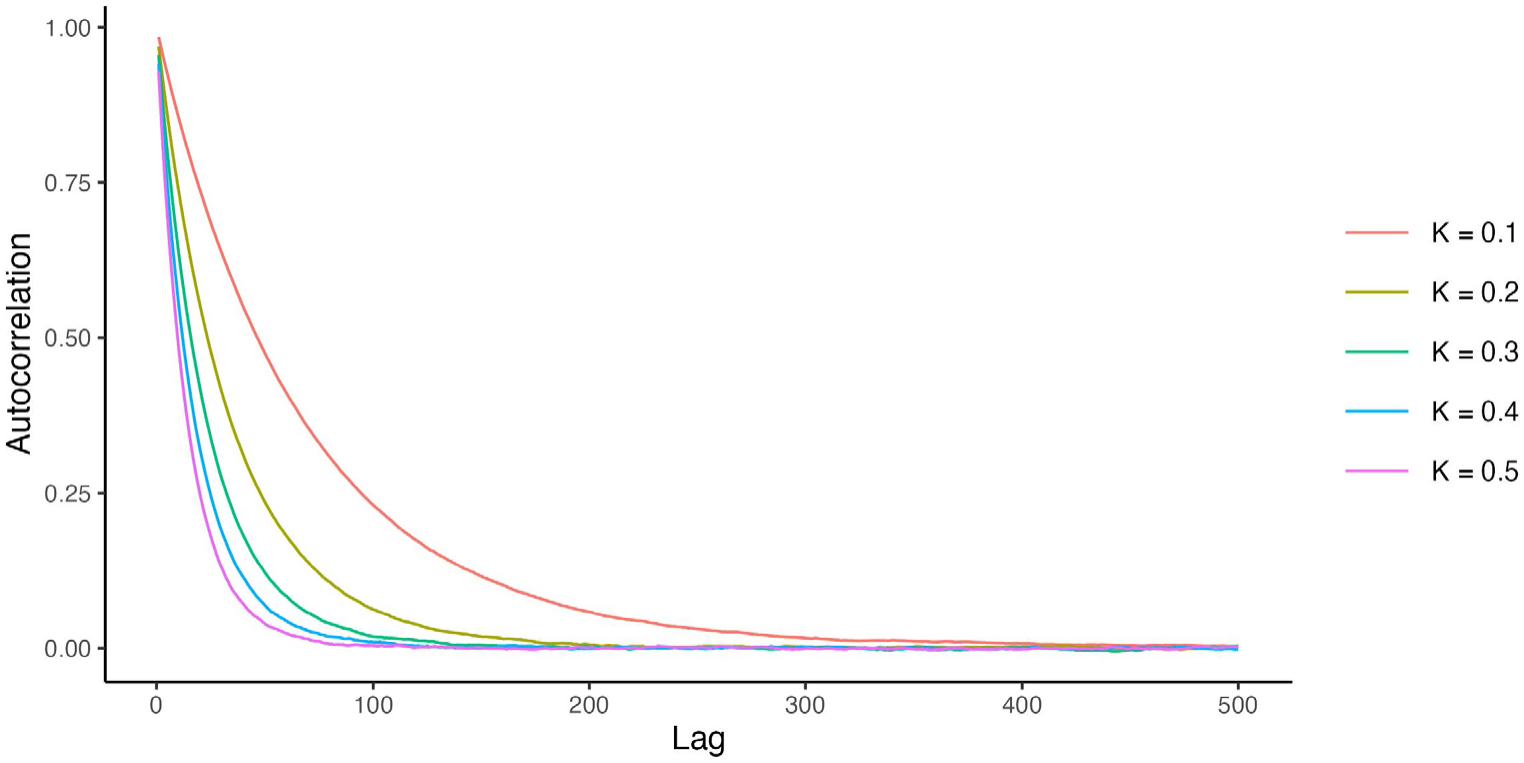
Autocorrelation of the Elo ratings at different lags l (i.e., the correlation between the Elo ratings of person j at t=1000 and t=1000−l across replications, averaged across persons), in the updated‐random scenario in conditions with different K‐values.

To remove rating variance inflation, we need to remove the dependence of item selection on the current errors, while keeping them dependent on the true values of ability and difficulty. One way to do this is to use ratings from previous timepoints instead of current ones. The problem, however, is that Elo ratings have very high autocorrelation. For example, Figure 8 shows autocorrelation of the ability ratings in the updated‐random scenario. Since we have multiple replications of the same system, we can examine the within‐person correlation between the ratings of that person from different replications at t=1000 and t=1000−l for various values of the lag l. For small l autocorrelation was very high (especially for large K). Thus, one often needs to go over a hundred responses back to find ratings independent of current ones,^5^ which is impractical since a person's true ability may have changed.

We propose an alternative approach which we will refer to as parallel Elo. Instead of having one rating value per person/item, we propose to have two ratings which are updated independently (denoted by ${\hat{\theta }}_{jt0}$ and ${\hat{\theta }}_{jt1}$ for persons, and ${\hat{\delta }}_{it0}$ and ${\hat{\delta }}_{it1}$ for items): ${\hat{\theta }}_{jt0}$ and ${\hat{\delta }}_{it0}$ are updated based on all odd responses, and ${\hat{\theta }}_{jt1}$ and ${\hat{\delta }}_{it1}$ are updated based on all even responses. Importantly, when ${\hat{\theta }}_{jt0}$ is updated item selection is done using ${\hat{\theta }}_{jt1}$ and vice versa. More formally, the algorithm is as follows:

Step 1. Select an item:

(6) $$i∼p(I_{jt}\;|\;{\hat{\theta }}_{jt(m_{jt}\;\mathrm{mod}\;2)},{\hat{\delta }}_{t(m_{jt}\;\mathrm{mod}\;2)})$$

Step 2. Update the ratings:

(7) $$\left[\begin{array}{c} {\hat{\theta }}_{j(t+1)0}\\ {\hat{\theta }}_{j(t+1)1} \end{array}\right]=\left[\begin{array}{c} {\hat{\theta }}_{jt0}\\ {\hat{\theta }}_{jt1} \end{array}\right]+\left[\begin{array}{c} \left(m_{jt}\;\mathrm{mod}\;2)K(X_{jit}-ℰ(X_{jit}\;|\;{\hat{\theta }}_{jt0},{\hat{\delta }}_{it0})\right)\\ \left(1-(m_{jt}\;\mathrm{mod}\;2))K(X_{jit}-ℰ(X_{jit}\;|\;{\hat{\theta }}_{jt1},{\hat{\delta }}_{it1})\right) \end{array}\right];$$

(8) $$\left[\begin{array}{c} {\hat{\delta }}_{i(t+1)0}\\ {\hat{\delta }}_{i(t+1)1} \end{array}\right]=\left[\begin{array}{c} {\hat{\delta }}_{it0}\\ {\hat{\delta }}_{it1} \end{array}\right]-\left[\begin{array}{c} \left(m_{jt}\;\mathrm{mod}\;2)K(X_{jit}-ℰ(X_{jit}\;|\;{\hat{\theta }}_{jt0},{\hat{\delta }}_{it0})\right)\\ \left(1-(m_{jt}\;\mathrm{mod}\;2))K(X_{jit}-ℰ(X_{jit}\;|\;{\hat{\theta }}_{jt1},{\hat{\delta }}_{it1})\right) \end{array}\right],$$

where $m_{jt}$ is the number of responses person j had before timepoint t.^6^ Since the ratings ${\hat{\theta }}_{0}$ and ${\hat{\theta }}_{1}$ are updated based on conditionally independent responses, the ratings are also conditionally independent. Notably, only using parallel item ratings is not sufficient since if parallel item ratings are updated alongside a single set of person ratings, they will not be conditionally independent and variance inflation will remain. While for item selection, the two ratings need to be used separately, for other purposes (e.g., providing feedback to students or teachers) they can be combined by taking the average.

## 4 SIMULATION STUDY WITH PARALLEL ELO

To demonstrate the properties of the parallel Elo ratings, we performed a simulation study. First, we repeated the updated‐adaptive scenario with parallel Elo instead of standard Elo. Here we are interested in the same research questions as formulated in the simulation setup of the main study. The same conditions as in the main study were included. For comparability, the number of responses was doubled compared to the simulation with standard Elo, since for each rating only half of the responses are used. Based on preliminary analysis, the number of item pairs was further increased in some of the conditions to ensure that convergence was reached before the last 500 item pairs: We used 1100 pairs of responses for the baseline and for p=.8, 2000 pairs for K=0.1, 1300 pairs for K=0.2, 1600 pairs for p=.9.

Second, we investigated how performance of parallel Elo compares to that of another rating system, the Urnings (Bolsinova, Brinkhuis, et al., 2022; Bolsinova, Maris, et al., 2022; Hofman et al., 2020), which was specifically designed to solve the issue of variance inflation. The comparison is not only interesting due to the similarities in focus of the rating systems, but in addition, Urnings generally require many responses to reach convergence. Partly, this is because of their discrete and stochastic nature, and parallel Elo effectively uses only half of the data. The speed of convergence is therefore interesting to compare. Comparing the two algorithms is quite involved, as we will show below. Therefore, we focus on a simple comparison of the baseline condition. Details on Urnings and the simulation setup for this comparison are provided below.

The Urnings rating system is similar to the ERS in that the ratings are updated after each response using a simple formula. There are, however, important differences. First, the parameters of the Rasch model are expit‐transformed: $\theta_{j}^{*}=\frac{1}{1+\exp(-\theta_{j})};$ and $\delta_{i}^{*}=\frac{1}{1+\exp(-\delta_{i})}$. Second, $\theta_{j}^{*}$ and $\delta_{i}^{*}$ are estimated using discrete ratings defined on a grid of proportions ${\hat{\theta }}_{jt}^{*}\in \{\frac{0}{n},\frac{1}{n},\text{…},\frac{n}{n}\}$, where $n\in {\mathbb{N}}^{+}$ is the urn size. Third, in the updating formula instead of the expected outcome $ℰ(X_{jit})$ we use a simulated outcome $X_{jit}^{*}$:

(9) $${\hat{\theta }}_{j(t+1)}^{*}={\hat{\theta }}_{jt}^{*}+\frac{1}{n}(X_{jit}-X_{jit}^{*});$$

(10) $${\hat{\delta }}_{i(t+1)}^{*}={\hat{\delta }}_{it}^{*}-\frac{1}{n}(X_{jit}-X_{jit}^{*});$$

where $X_{jit}^{*}$ is generated under the Rasch model with $\frac{{\hat{\theta }}_{jt}^{*}+X_{jit}}{n+1}$ and $\frac{{\hat{\delta }}_{it}^{*}+1-X_{jit}}{n+1}$ (transformed back to the logit scale) instead of the true values. The inverse of the urn size serves a similar role as the K‐value in Elo. After an update, the rating can go up ($X_{jit}^{*}<X_{jit}$) or down ($X_{jit}^{*}>X_{jit}$) by $\frac{1}{n}$, or stay constant ($X_{jit}^{*}=X_{jit}$). Fourth, to avoid rating variance inflation caused by adaptive item selection, updates are accepted with a probability equal to the ratio between the probability of item i being selected for person j after and before the update (i.e., the Metropolis‐Hastings algorithm is used to restore the detailed balance of the chain of the ratings which is distorted by adaptivity). These differences from Elo ensure good theoretical properties of Urnings: the joint invariant distribution of all ratings multiplied by n is a product of binomial distributions with $\theta^{*}$s and $\delta^{*}$s as the probability parameters and n as the number of trials, conditional on their total sum. In a large system, the marginal distributions are very close to independent binomials, hence the parameters are unbiased and their variances are known.

To compare Urnings and parallel Elo in terms of convergence speed we need to make them comparable in terms of variance of the ratings, as speed can always be increased by increasing step size and variance. This is, however, not trivial because when transforming parameters from the logit scale to the [0,1] scale and back the step size is not constant across the scale (e.g., a step of $\frac{1}{n}$ close to .5 is smaller on the logit scale than the same step close to 0 or 1). To choose an urn size, we matched the reliability (squared correlation between $\theta^{*}$ and ${\hat{\theta }}^{*}$) and the overall accuracy (mean squared error of ${\hat{\theta }}^{*}$ averaged across persons^7^) of the ratings upon convergence in two systems (for urnings they can be calculated based on the known invariant distributions without simulating a learning system), resulting in n=32 for the baseline condition. However, this does not guarantee that the accuracy of each individual rating upon convergence would be the same across systems.

We ran the Urnings algorithm for systems with 2,200 item responses with items selected adaptively with p=.7 (matching the baseline condition). For item selection, we used $\frac{n\theta_{j(t-1)}^{*}+1}{n+2}$ and $\frac{n\delta_{i(t-1)}^{*}+1}{n+2}$ instead of the ratings themselves to prevent the selection probability in Equation 4 from becoming undefined for ratings of 0 or 1. The average ratings were computed for every timepoint, then transformed to the logit scale, and rescaled to have the same identification constraint as the Elo ratings ($\sum_{i}{\hat{\delta }}_{it}\equiv 0$). To avoid an involved comparison between these two rating systems, we limited ourselves to the baseline condition.

### 4.1 Simulation results

**FIGURE 9:**
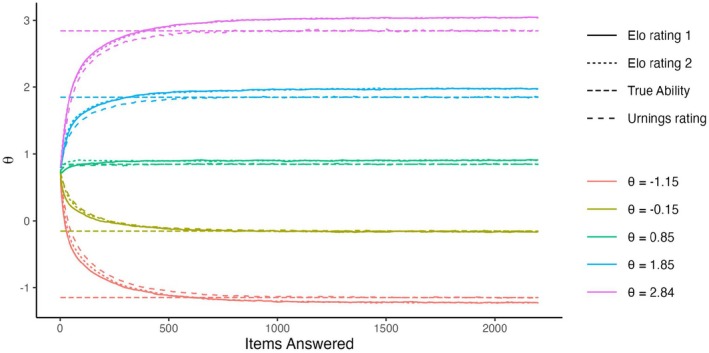
The means of the ratings from parallel Elo (‘Elo Rating’ and ‘Elo Rating 2’ refer to the two parallel ratings, ${\hat{\theta }}_{0}$ and ${\hat{\theta }}_{1}$) and Urnings of five persons with different true values of ability (θ) at different timepoints in the baseline condition of the updated‐adaptive scenario.

**FIGURE 10:**
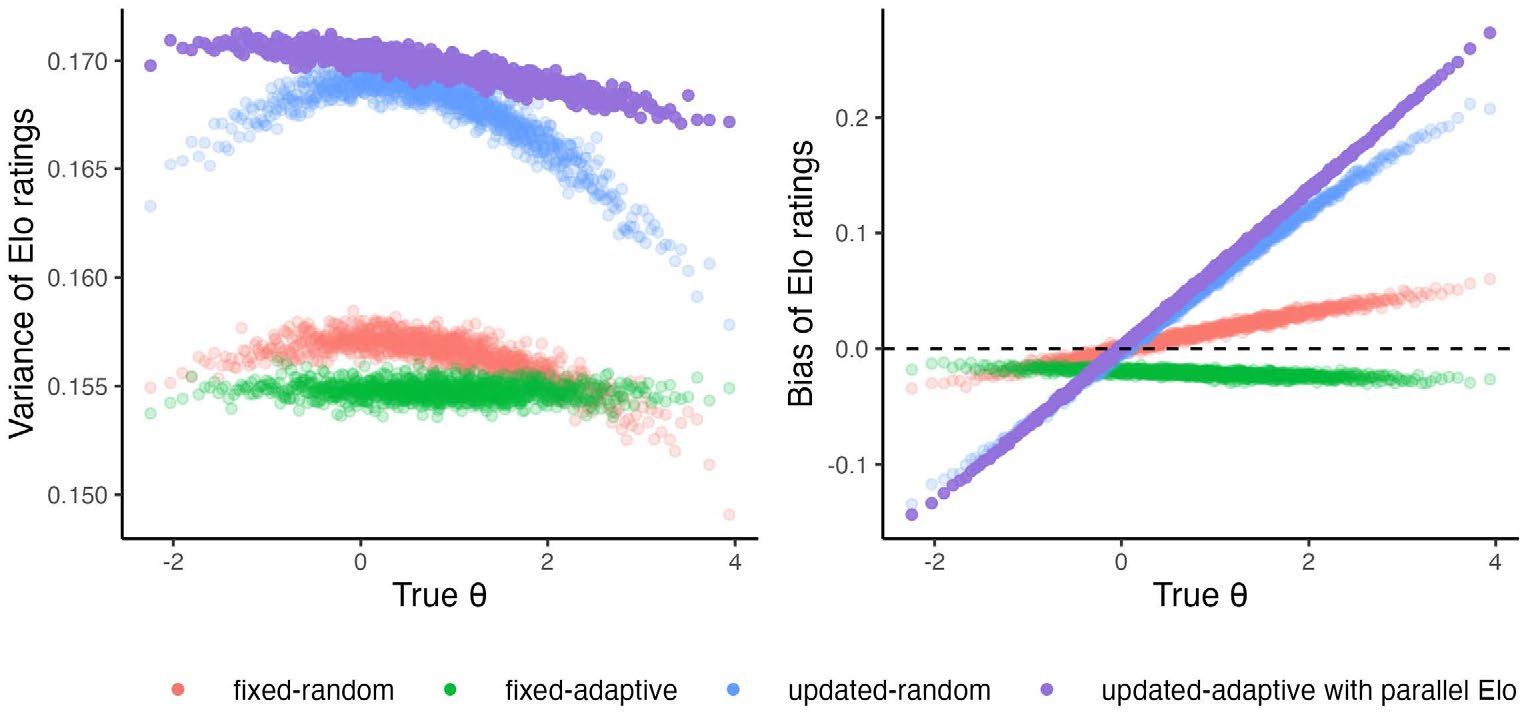
Variance (on the left) and bias (on the right) of the Elo ratings as the function of the true values of ability (θ) in the baseline conditions of the updated‐adaptive scenario with parallel Elo compared to the other scenarios with standard Elo.

**FIGURE 11:**
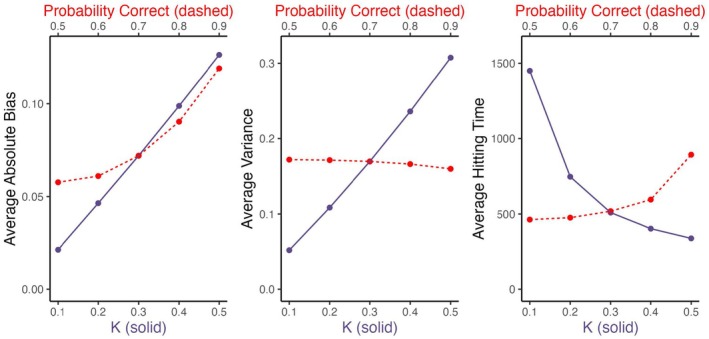
Effects of the K value (blue, solid) desired probability of a correct response (p; red, dashed) on the average absolute bias (left), average variance (middle), and average hitting times (right) of the Elo ratings in the updated‐adaptive scenario with parallel Elo.

Figure 9 shows that unlike with standard Elo, with parallel Elo the ratings converge to an invariant distribution in the updated‐adaptive scenario. Like in the other scenarios (with standard Elo) the ratings are biased. In Figure 10 on the right, we see outward bias of the ratings (average absolute bias of .072 compared to .064 in the updated‐random scenario). The variance of the Elo ratings was on average slightly larger (.170) than that in the updated‐random scenario (.168). Figure 10 (on the left) shows how the variance of the Elo ratings depends on the true values. The effect is a lot weaker than in the updated‐random condition, but still, for the persons in the tails of the ability distribution the variance is smaller than for those close to the mean of item difficulty. Figure 11 shows that K and p have the same effects on the properties of the invariant distributions as in the other simulation scenarios (see Figures 3 and 4 for comparison).

Figure 9 also shows how Urnings of specific persons evolve over time. While Elo ratings are biased, the Urnings converge to the true values. The Elo ratings move faster in the direction of the true values compared to Urnings, but since their invariant means are further away from the starting point, it takes longer to reach the invariant mean (average hitting times for Elo and Urnings were 512.2 and 456.1, respectively, which is about 5 times higher than in the updated‐random scenario^8^). Performance of parallel Elo in terms of the amount of data needed for accurate measurement is, hence, quite close to Urnings, and more research is needed to pinpoint under which conditions either of the methods should be preferred.

## 5 DISCUSSION

We start from summarising our main results on the measurement properties of the standard ERS which had not been previously shown in the literature: (1) In the random scenarios, the Elo ratings have outward bias, while in the fixed‐adaptive scenario, bias is negative for p>.5. (2) The variance of the ratings in the random scenarios decreases as ability moves away from the average item difficulty (opposite to the standard errors of ability in standard item response theory). (3) The variance of the ratings in the fixed‐adaptive scenario hardly depends on p, but achievable measurement precision is indirectly effected through slower convergence for p>.5.

While the phenomenon of rating variance inflation had been shown previously, in this study we have not only demonstrated that in the updated‐adaptive scenario, common in adaptive learning systems, the ratings diverge after every response, but also investigated the factors effecting the speed of this divergence and showed why it happens. In educational applications, where the ERS seems to be the go‐to algorithm for on‐the‐fly calibration and adaptivity, these findings may impact many implementations.

In practice, the size of the impact can be limited. In our simulation, we used a constant K‐value both for the persons and for the items. In many applications, however, a decreasing K‐value is often used, and especially for the items, it can become very small. In practice, items might therefore come close to being fixed, which could explain why the severity of variance inflation in real data is not as large as in our simulation. See for example Hofman et al. (2020), who show an increase of standard deviation of ability of about 3% after four years. Moreover, in many low‐stakes applications, not the *ratings* but the *rankings* of the abilities and difficulties being preserved is sufficient, limiting the severity of the impact of our findings in certain contexts.

For applications with higher stakes and that do involve measurement, for example applications that combine learning and assessment, mere rankings are not sufficient, and measurement properties cannot be ignored. To meet this need, a more formal and easy‐to‐implement solution has been presented, where this variance inflation can be fixed by introducing parallel chains of independent Elo ratings. Additionally, with parallel Elo one can quantify the reliability of the ratings by computing the correlation between ${\hat{\theta }}_{0}$ and ${\hat{\theta }}_{1}$. However, with parallel Elo in the updated‐adaptive scenario the bias and variance of the ratings and the number of responses needed to reach convergence are larger than in the other scenarios (with the standard ERS). Compared to the Urnings rating system, with parallel Elo less responses are needed for the ratings to get close to the true values, but more responses are needed for them to stabilize and reach their invariant distribution because of the presence of outward bias.

Our approach also includes several limitations. The simulation conditions have been specifically chosen to be ecologically valid, yet there are always situations that have not been simulated. For example, in practice, item banking includes exposure control, heterogeneity in the distribution of item difficulties, local dependence, etc. Also, we simplified simulations by using the same generating model as was used for estimation and disregarding complexities in real learning systems that might include all sorts of dynamic changes. We expect that some of these complexities would lead to worsened performance of Elo, while others (e.g., exposure control) would reduce some of these problematic effects.

Concerning future work, one direction is to test the parallel chains approach in practical applications and further compare it with existing approaches. Another direction is to further explore the bias of the Elo ratings. Since this bias is generally linear, the utility of post‐hoc bias corrections can be considered in the future. Similarly, based on the simulation results, upper bounds for the standard errors of Elo ratings could be derived. However, with these improvements in ERS (together with the implementation of parallel Elo), only approximations to the invariant distributions would be available. If one prefers to have unbiased ratings with known distributions, then alternatives, such as the Urnings Bolsinova, Maris, et al. (2022) can be considered. At the same time, there are practical reasons to prefer using the Elo system with the proposed improvements. Compared to Urnings, the ERS is easier to implement and to communicate to stakeholders, and it provides estimates on the usual logit scale. Furthermore, the Elo update allows for more flexibility in terms of measurement models. An interesting direction for future research is to explore hybrid methods combining the ERS with Urnings, for example using ERS to escape a cold start quicker and switching to Urnings to obtain unbiased ratings with known standard errors. With knowledge of Elo's measurement properties and with the proposed improvements for it, we believe that the ERS can certainly be kept alive and remain being well‐used in educational applications.

## AUTHOR CONTRIBUTIONS

**Maria Bolsinova:** conceptualization; methodology; software; formal analysis; investigation; validation; funding acquisition; visualization; project administration; writing – review and editing; writing – original draft; data curation. **Bence Gergely:** conceptualization; methodology; visualization; software; formal analysis; writing – review and editing. **Matthieu J. S. Brinkhuis:** conceptualization; methodology; writing – review and editing.

## CONFLICT OF INTEREST

The authors declare that there are no conflicts of interest related to this article.

## Supporting information


Data S1.

## Data Availability

The data that support the findings of this study are openly available in Open Science Framework (OSF) repository at https://osf.io/yg8p6.

## Footnotes

^1^
Extensions of the algorithm allow to, for example, estimate more complex models (e.g., models for polytomous items (Zhang et al., 2023), multidimensional item response theory (Park et al., 2019), models which incorporate response times (Klinkenberg et al., 2011)), include a dynamic K‐value (e.g., Glickman, 1999), etc.

^2^
The code repository to replicate the simulations and all the presented material in this paper, can be found at: https://github.com/mrpogge/ELO_distribution?tab=readme‐ov‐file. The data from the simulations is also available at: https://osf.io/yg8p6/.

^3^
In preliminary simulations we also included conditions in which random error was added to the fixed item difficulties. The results were very similar to when the true value were used, therefore we proceeded with true values.

^4^
To obtain higher precision of the estimates of the person‐specific means and variances we ran 10,000 replications instead of 500 in the baseline conditions.

^5^
In Data S1 we show a small simulation in which we varied the lag used for item selection to examine its effect on the presence and severity of rating variance inflation in the updated‐adaptive scenario.

^6^
Alternatively, one could define even and odd responses based on the total number of responses in the system before the response of person j at timepoint t. We do not prefer this alternative because while it requires one to keep track of only one counter, it does not guarantee that for each person the ability rating used for item selection is the most recent one.

^7^
Reliability and mean squared error had to be computed for the Elo ratings transformed to the [0,1] scale because the boundary values of the ratings from Urnings are undefined on the logit scale.

^8^
Since we observed that when the system is initiated from a cold start and item selection is directly adaptive, convergence is very slow, we conducted an additional simulation, where only 20% of the persons the ratings were initiated from a cold start, while the ratings of the items and the rest of the persons were taken from a converged system, see Data S1 for details.
