# Supplementary material for: Keeping Elo alive: Evaluating and improving measurement properties of learning systems based on Elo ratings
Source: Br J Math Stat Psychol. 2025 Jun 6;79(1):95–110. doi: 10.1111/bmsp.12395 (PMC12784335; doi:10.1111/bmsp.12395)
Supplement: Supplementary file 1 — Data S1. [file BMSP-79-95-s001.pdf]

Supplementary materials

Simulation with lagged item selection

We repeated the simulation in the baseline condition ($K = 0.3, P = .7$) of the updated-adaptive scenario with lagged adaptive item selection. We varied the lag which was used for item selection to examine its effect on the presence and severity of rating variance inflation, $l = \{1, 2, 5, 10, 20, 50, 100\}$. In the first l timepoints the items were selected randomly after which the values at timepoint $(t - l)$ were used for item selection. Since adaptive item selection started only at the $(l + 1)$th timepoints we increased the number of timepoints to $(1000 + l)$.

Figure 1 shows that the severity of variance inflation decreases with larger lags, but for most l it is still present. For $l = 100$, there is no variance inflation and the ratings stabilise. We further inspect the properties of the invariant distributions of the ratings for $l = 100$. The ratings converge to values different from true values with an outward bias (average absolute bias was 0.076) which was larger than that of the updated-random scenario (0.064). The variance of the Elo rating was on average slightly larger (0.169 with lagged item selection) than that in the updated-random scenario (0.168).

Post-hoc simulation with new persons in a functioning learning system

Since we observed that when the system is initiated from a cold start and item selection is directly adaptive, convergence is very slow, we conducted an additional simulation, where only 20% of the persons the ratings were initiated from a cold start, while the ratings of the items and the rest of the persons were taken from a converged system. The idea was to evaluate how fast the ratings of new persons entering an already functioning learning system converge. This post-hoc simulation was performed for the baseline condition of the updated-random scenario with standard Elo, and of the updated-adaptive scenario with parallel Elo and Urnings. To specify the startings values, for (parallel) Elo we first ran a system for 1100 (pairs of) responses from a total cold start and used the last ratings of the items and 80% of the persons as starting values in this post-hoc simulation. While for Urnings we sampled the starting values

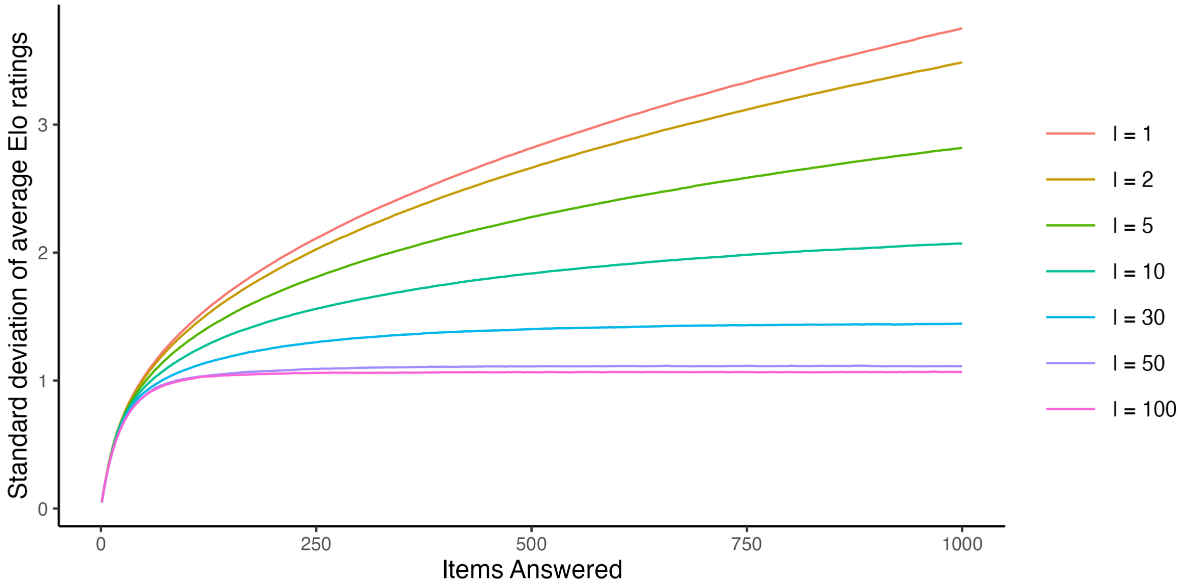

Figure 1

Standard deviation of the average Elo ratings over time for lagged item selection with different lags (l).

from the theoretical invariant distribution.

In the updated-random scenario the effect of a converged system was not large: 94.7 items instead of 102.6 were needed on average. In the updated-adaptive scenario the effect was much stronger: Average hitting times were roughly halved (243.2 for parallel Elo and 230.6 for Urnings). That is, for the new persons entering a calibrated system only about 2.5 more responses are needed for convergence when items are selected adaptively compared to when they are selected randomly.
